# Physicians’ Perceptions as Predictors of the Future Use of the National Death Information System in Peru: Cross-sectional Study

**DOI:** 10.2196/34858

**Published:** 2022-08-15

**Authors:** Javier Vargas-Herrera, Giovanni Meneses, Juan Cortez-Escalante

**Affiliations:** 1 Department of Preventive Medicine and Public Health National University of San Marcos Lima Peru; 2 Pan American Health Organization Brasilia Brazil

**Keywords:** death certificates, health information system, mortality, vital statistics, Technology Acceptance Model, model, acceptance model, certificates, information system, physicians, predictors, cross-sectional study, analysis, death

## Abstract

**Background:**

A computer application called the National Death Information System (SINADEF) was implemented in Peru so that physicians can prepare death certificates in electronic format and the information is available online. In 2018, only half of the estimated deaths in Peru were certified using SINADEF. When a death is certified in paper format, the probability being entered in the mortality database decreases. It is important to know, from the user’s perspective, the factors that can influence the successful implementation of SINADEF. SINADEF can only be successfully implemented if it is known whether physicians believe that it is useful and easy to operate.

**Objective:**

The aim of this study was to identify the perceptions of physicians and other factors as predictors of their behavioral intention to use SINADEF to certify a death.

**Methods:**

This study had an observational, cross-sectional design. A survey was provided to physicians working in Peru, who used SINADEF to certify a death for a period of 12 months, starting in November 2019. A questionnaire was adapted based on the Technology Acceptance Model. The questions measured the dimensions of subjective norm, image, job relevance, output quality, demonstrability of results, perceived usefulness, perceived ease of use, and behavioral intention to use. Chi-square and logistic regression tests were used in the analysis, and a confidence level of 95% was chosen to support a significant association.

**Results:**

In this study, 272 physicians responded to the survey; 184 (67.6%) were men and the average age was 45.3 (SD 10.1) years. The age range was 24 to 73 years. In the bivariate analysis, the intention to use SINADEF was found to be associated with (1) perceived usefulness, expressed as “using SINADEF avoids falsifying a death certificate” (*P*<.001), “using SINADEF reduces the risk of errors” (*P*<.001), and “using SINADEF allows for filling out a certificate in less time” (*P*<.001); and (2) perceived ease of use, expressed as “I think SINADEF is easy to use” (*P*<.001). In the logistic regression, perceived usefulness (odds ratio [OR] 8.5, 95% CI 2.2-32.3; *P*=.002), perceived ease of use (OR 10.1, 95% CI 2.4-41.8; *P*=.001), and training in filling out death certificates (OR 8.3, 95% CI 1.6-42.8; *P*=.01) were found to be predictors of the behavioral intention to use SINADEF.

**Conclusions:**

The behavioral intention to use SINADEF was related to the perception that it is an easy-to-use system, the belief that it improves the performance of physicians in carrying out the task at hand, and with training in filling out death certificates.

## Introduction

Medical death certification is the main source of information on causes of death in a population [1]. Various studies worldwide report that there is low coverage of deaths that have medical certification of death [2,3], and those deaths that have certification of the causes of death do not have the desired quality [4,5]. In 2016, the coverage of deaths with medical certification of the causes of death in Peru was 56% [6] and the quality, measured as the proportion of codes of causes of death in the International Classification of Diseases classified as “garbage code,” was among the highest in the world [7]. In 2017, a computer application called the National Death Information System (SINADEF) [8] was implemented in Peru, which is used by physicians to prepare death certificates online. This system was the result of coordinated work between the National Institute of Statistics and Informatics, the National Registry of Identification and Civil Status, and the Ministry of Health, supported by the Bloomberg “Data for Health” initiative. Before implementing SINADEF, physicians performed death certification on paper forms, which were entered into computer applications installed on computers with local databases, which were then sent through email messages at the regional and national levels. In addition to the lack of opportunity for data availability, this system has many other problems: it does not verify the identity of the deceased by consulting a database, it does not identify the hospital where the person died, it does not unequivocally locate the district where the death occurred, and often, the cause of death written by the doctor is illegible.

With SINADEF, physicians can certify a death online, verify the identity of the deceased and the hospital where the death occurred, consult online databases, and more accurately record the place of death. Further, the problem of the lack of legibility of the doctor’s handwriting, which is very important in the registry of causes of death, disappears. From the perspective of electronic government, information and communication technology (ICT) can facilitate the services provided to citizens. It allows health authorities at all levels of government to monitor mortality indicators in a timely manner and improve prevention and control actions, as is happening now with the monitoring of the COVID-19 pandemic. Likewise, it allows other state agencies to be more efficient in public policy management processes. These aspects justify taking the greatest precautions to guarantee its successful implementation.

A paper [6] that reported the first results of the implementation of SINADEF showed that in July 2018, only half of the estimated deaths were certified using that system, and it identified some gaps and barriers that could limit the achievement of adequate coverage and quality of the registry of causes of death. One of them is the modality used by the doctor to certify a death [6]. When a doctor certifies a death on paper forms, the data may not enter the system. The paper forms can remain with the relative of the deceased or in a drawer of a hospital office, in funeral agencies, in the civil registry, etc. In July 2018, half of the estimated deaths were not entered in SINADEF. Some studies about the map of death registration processes carried out in Peru indicate that deaths registered in paper format are less likely to enter the mortality database [9], which highlights the importance of identifying factors linked with physicians, thereby improving the use of SINADEF to certify a death. Furthermore, SINADEF has played a fundamental role in documenting the excess mortality that occurred during the COVID-19 pandemic in Peru. Several studies [10-12] used SINADEF to warn, from the beginning of the first wave of transmission of COVID-19 in Peru, a significant difference in deaths that the physicians who used SINADEF attributed to COVID-19 and that were not reported by the system of epidemiological surveillance. In addition, they coincide in pointing out the importance of the quality of the data provided by SINADEF and the need to strengthen the entire death registration process so that it is useful for containing the health emergency. Recent studies using SINADEF reported that excess mortality from all causes in 2020 was more than 100,000 deaths and that more realistic figures have been obtained owing to coordination initiatives between those responsible for epidemiological surveillance, recording of vital events, and diagnosis [13]. However, they did not notice that a significant proportion of deaths, especially those that occur in the community, are not reported through SINADEF; therefore, it is important to evaluate the factors that could limit its real use.

Intuitively, it may seem that the information production process supported by paper format, when replaced by an electronic registry, as is the case with SINADEF, will immediately produce obvious improvements such as the reduction of errors due to data transcription. However, there are also bad experiences in the implementation of ICT projects in health services, which in theory were very robust, probably because the user experience was not considered in the design of the applications [14]. Therefore, it is necessary to know the determinants of the future use of a technology.

All innovation in health requires the intensive participation of the people who will be affected by the processes in which it will intervene. To expand the use of online death certification, it is important to know what the physicians think about the system. Some barriers related to the system itself are dependence on internet connectivity, limited availability of computers and printers in hospitals, and failures in technical support and application maintenance. Although the advantages include their use even from mobile devices, physicians may not be willing to use them. To understand why users accept or use new technologies, a predictive evaluation methodology of technology use known as the Technology Acceptance Model (TAM) has been proposed, which has been tested and validated in different contexts and studies [15,16]. The TAM has been used in the evaluation and implementation of ICT in the health field [17-20]. The TAM is based on user perceptions. If people believe that an ICT application helps them to do their job better (“perceived usefulness”) and, at the same time, that it does not require additional effort (“perceived easy to use”), they will end up adopting that technology [15].

Rahimi et al [21] conducted a systematic review of 134 studies published between 1999 and 2017 that used TAM to evaluate ICT applications in the health field after their initial introduction. The areas of ICT studied were general information technologies, health information systems, electronic health records or electronic medical records, electronic prescription systems (e-prescription), pocket computers, telemedicine, mobile health, and personal health records. An online system for medical certification of death can be considered an electronic medical record component, but in this review, no study specifically evaluated an information system like SINADEF. The studies evaluated are widely distributed throughout the world, but they are scarce in Latin America, and none were reported in Peru. The main findings were that the application of TAM was quite heterogeneous. Most studies used extensions of the original TAM, which suggests that there is no optimal version of TAM to use in the field of health. Holden and Karsh [16] also carried out a review of TAM and highlight the importance of developing “the left part of the model,” that is, the determinants with specific, contextualized, and actionable constructs.

In Peru, we have not identified previous research that describes the use of TAM to evaluate ICT in health; only 1 study evaluated the intended use of mobile banking services [22]. Our study aims to identify the perceptions of physicians about the usefulness and ease of use of SINADEF and other factors such as predictors of the behavioral intention to use SINADEF, contextualizing the predictor variables with specific propositions about the use of SINADEF to certify deaths.

## Methods

### Design

An observational, cross-sectional study was carried out that consisted of the application of a survey directed to physicians who used the national computer system of deaths (SINADEF) to produce a death certificate. The study population was made up of physicians with a professional practice in Peru and who, as of 2017, had the chance to carry out, at least once, the death certification process online through SINADEF.

### Recruitment

Between November 24, 2019, and November 18, 2020, an electronic form was sent to 26,185 physicians who were registered as SINADEF users until December 31, 2017. Of them, 505 physicians opened the electronic form and 424 agreed to participate in the study; 111 physicians were excluded because they reported that they had no experience in the use of SINADEF, and 41 physicians were excluded because they had contradictory answers about their degree of agreement with the use of SINADEF to certify a death. Therefore, the sample consisted of 272 physicians.

### TAM

#### Questionnaire

The questions in the questionnaire were based on TAM. TAM proposes that the behaviors of individuals depend on their beliefs and subjective norms and that the use of a new technology depends on 2 variables in particular: the perceived ease of use of the technology and the perceived usefulness. In turn, these 2 variables will directly influence the attitude of the individual toward the actual use of technology [15]. In a simpler way, if an individual has the belief that a technology is easy to use and at the same time, it is useful for the work that it has to fulfill, he or she will have a favorable attitude to use the technology effectively. In this study, the TAM2 model proposed by Venkatesh and Davis [23] was used, which incorporates the following variables: subjective norm, image, job relevance, output quality, and results demonstrability. The measurement scale used was based on the original proposals of Venkatesh and Davis [23] for the evaluation of information technology in general but adapted to the context of its use in the medical certification of deaths in Peru, following the recommendations of Holden and Karsh [16] on adapting the model specifically to the health context by using belief induction methods. In the propositions of beliefs, reference is made to the regulatory provisions of the Ministry of Health in Peru or the comparison of the use of SINADEF with the traditional way of using paper forms. The following operational definitions were used for each of the explanatory variables proposed in the model used (Textbox 1).

In addition, variables external to the model are included that can be explanatory or that intervene in the attitude or behavior of intention to use the technology of physicians, such as age, sex, medical specialty, workplace, time spent in service, and training in the filling of death certificates.

**Operational definitions of the Technology Acceptance Model used.**

**Subjective norm:** Subjective perception of the individual on social pressures, which includes the perception of the beliefs of relevant people, for the adoption of the behavior of use of the National Death Information System (SINADEF), through their opinion of agreement or disagreement on a Likert scale of the following propositions:

- “I have to use SINADEF to certify deaths because it’s already established that way.”
- “The authorities of my hospital and the Ministry of Health could sanction me if I do not use SINADEF to certify deaths.”
- “I have to use SINADEF because everyone already uses it to certify deaths.”

**Image:** Refers to the user’s self-perception, visual, or mental representation produced by the use of SINADEF. It was measured by their opinion of agreement or disagreement on a Likert scale of the following propositions:

- “I feel comfortable with information and communication technologies.”
- “I consider myself a person open to change.”
- “I have good adaptability.”

**Job relevance:** Refers to the perceived attribution of the user that SINADEF is important for the performance of work tasks. It was measured by their opinion of agreement or disagreement on a Likert scale of the following propositions:

- “When SINADEF is implemented, the causes of death of the population will be known in a timelier manner.”
- “The use of SINADEF will help the directors or managers of health organizations make decisions.”
- “If SINADEF is consolidated, it will be possible to quickly consult the data of the deceased.”

**Output quality:** Refers to the real or perceived attribution of the SINADEF user of the quality of the report provided by the technology. It was measured by their opinion of agreement or disagreement on a Likert scale of the following proposition:

- “The SINADEF death certificate is of higher quality than the paper format.”

**Perceived usefulness:** Refers to an individual’s perception that the use of SINADEF will improve job performance. It was measured by their opinion of agreement or disagreement on a Likert scale of the following propositions:

- “Using SINADEF avoids falsifying a death certificate.”
- “Using SINADEF reduces the risk of errors in death certificates.”
- “Using SINADEF allows me to produce a death certificate in less time than doing it in paper format.”

**Perceived ease of use:** Refers to an individual’s perception that using SINADEF does not require effort, through their opinion of agreement or disagreement on a Likert scale of the following propositions:

- “I think SINADEF is easy to use.”
- “It’s easy to get a password to use SINADEF.”
- “It’s easy to recover the SINADEF password when you forget it or it’s blocked.”
- “It’s easy to get technical support from SINADEF when you need it.”

**Behavioral intention to use:** Refers to the motivation or willingness of an individual to make the effort to use SINADEF to certify a death, through their opinion of agreement or disagreement on a Likert scale of the following proposition:

- “When I need to certify a death, I will use the SINADEF.”
- “In any circumstance, if I need to certify a death, I will not use SINADEF.”

**Results demonstrability:** Thinking that SINADEF allows you to demonstrate the results, through their opinion of agreement or disagreement on a Likert scale of the following proposition:

- “I can easily communicate the results of my experience using SINADEF.”
- “For me, the results of using SINADEF will be visible.”

#### Instrument Validation

The content of the proposed instrument was validated through an expert judgment process [24]. Six expert researchers were selected—2 of them experts in measurement and evaluation. The index of agreement of the judges on the propositions of the TAM variables was 84.9%, with a κ index of 0.7304. All elements with a κ index less than 0.6 were removed and replaced. Items with a κ index of 0.6571 were reviewed and their formulation was paraphrased or modified based on the judges’ recommendations. The reliability of the 20-item measurement scale for the 8 variables that were included in the study was evaluated with the Cronbach α coefficient. The value obtained for the Cronbach α coefficient was .874.

### Statistical Analysis

#### Overview

The data collected through the electronic forms were analyzed using the SPSS statistics package (IBM Corp). A description of the variables was made, presenting arithmetic means and standard deviations of the quantitative variables, such as age and years of medical work, and frequency tables of qualitative variables such as sex, workplace, and medical specialty. A bivariate analysis was performed between the independent variables such as perceived usefulness or perceived ease of use and the dependent variable behavioral intention to use. Subsequently, a multivariate analysis was performed using binary logistic regression with the Wald successive steps method with a likelihood ratio, evaluating models that included both descriptive and explanatory variables as independent variables, with the dependent variable behavioral intention to use. To carry out the bivariate analysis and binary logistic regression analysis, the responses to the propositions of each of the variables on a Likert scale were transformed into 2 alternatives: “agree” and “disagree.” In the initial model, all the variables of TAM that were found associated with the behavioral intention to use in the bivariate analysis were considered as independent or predictive variables. Additionally, the variables sex, age, main work center, and training in filling out death certificates were entered into the model. Following the TAM2 model, the variables subjective norm, image, job relevance, output quality, demonstrability of results, and perceived ease of use were crossed with perceived usefulness. In the successive steps, the variables with a Wald value less than 1 and with statistical significance less than .05 were eliminated.

#### Power

In the bivariate analysis, the chi-square test was used to establish the existence of an association between the variables, choosing a confidence level of 95%, a level of statistical significance of (α) equal to or less than .05, and a power of 80% (1-*β*) equal to or less than .20. To identify the variables associated with behavioral intention to use in the multivariate analysis, the logistic regression with Wald method (backward) of successive steps with the likelihood ratio was used. A confidence level of 95% and a significance level (α) equal to or less than .05 were chosen.

### Ethics Approval

The research protocol and the informed consent signed by the participants were approved by the Research Ethics Committee of the San Marcos University School of Medicine (#19-0027).

## Results

### Sample

In this study, 272 physicians, who were SINADEF users since 2017, were studied. Most (184/272, 67.6%) were male. The average age was 45.3 (SD 10.1) years. The minimum age was 24 years and the maximum age was 73 years. Most of the respondents (100/272, 36.8%) were in the age group of 40 to 49 years. Only among young physicians between the ages of 24 and 30 years did women predominate (10/17, 58.8%), while in the rest of the age groups, there was a predominance of male physicians.

### Description of the Population Studied

The most frequent participants in this study were general practitioners (70/272, 25.7%), pediatricians or neonatologists (30/272, 11%), internists (25/272, 9.2%), and obstetrician-gynecologists (15/272, 5.5%). Forensic physicians constituted 4.4% (12/272) of the participants. The study included graduates of all universities that have a faculty of medicine in Peru. Approximately 5.1% (14/272) of the participants studied abroad. Most of the study participants worked in hospitals as their main workplace, followed by those who worked in private clinics and health centers. Most of the participants (221/272, 81.3%) had worked for 10 or more years. Approximately 71% (193/272) stated that they had received some type of training in filling death certificates and among these, the majority did so through conferences given at the same hospital, through health services networks, or through a combination of training modalities (see Table 1).

**Figure 1:**
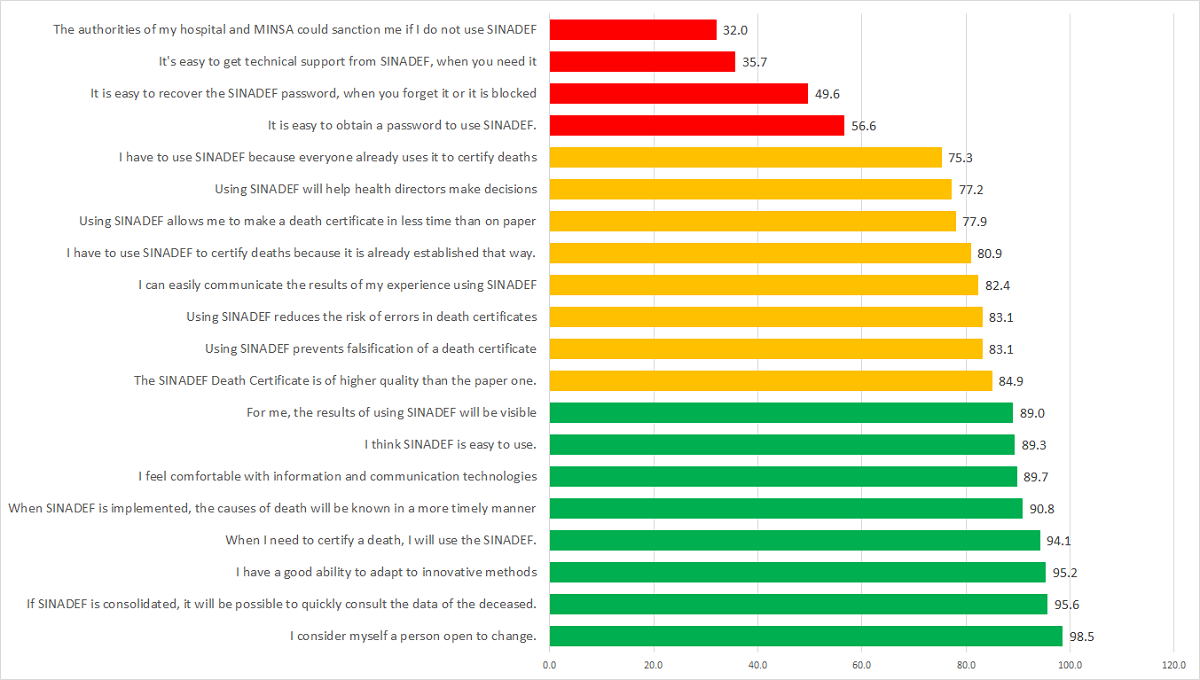
Level of agreement (%) in the physicians' perceptions about the use of the National Death Information System. MINSA: Ministry of Health; SINADEF: National Death Information System.

Figure 1 shows the perceptions classified by their level of agreement among the respondents, and those with the highest agreement were perceived as people open to change, who considered the usefulness of the system to quickly consult information about the deceased, and the ability to adapt to innovative methods. Those who had less agreement corresponded to the ease of obtaining passwords or technical support and the possible sanctions that their nonuse would cause.

**Table 1:** Description of the main characteristics of the studied population (N=272).

Characteristics		Values, n (%)
**Sex**		
	Male	184 (67.6)
	Female	88 (32.4)
**Age group (years)**		
	24-30	17 (6.3)
	30-39	60 (22.1)
	40-49	100 (36.8)
	50-59	70 (25.7)
	>59	25 (9.2)
**Main workplace**		
	Hospital	190 (69.9)
	Health center/post	32 (11.8)
	Private clinic/physician’s office	29 (1.7)
	Medical-legal division	16 (5.9)
	Other	5 (1.8)
**Years of medical work**		
	Less than 10	51 (18.8)
	10-19	99 (36.4)
	20-29	83 (3.5)
	More than 29	39 (14.3)
**Training in filling death certificate**		
	Yes	193 (71)
	No	79 (29)
**Training modality (n=193)**		
	A conference at the health center or through a health services network	58 (3.1)
	During undergraduate studies	28 (14.5)
	During postgraduate studies	20 (1.4)
	A virtual course	14 (7.3)
	Combined (more than one of the above)	73 (37.8)

### Bivariate Analysis

Physicians’ perceptions that were found to be associated with behavioral intention to use (*P*<.05) were perceived usefulness as shown by “using SINADEF avoids falsifying a death certificate,” “using SINADEF reduces the risk of errors,” and “using SINADEF allows for filling out a certificate in less time,” and perceived ease of use as shown by “I think SINADEF is easy to use,” as well as other perceptions related to image, job relevance, output quality, and results demonstrability. As a subjective norm, no proposition was found associated with behavioral intention to use (see Table 2). There was also no significant association between age, sex, occupation characteristics, or training in filling out death certificates with behavioral intention to use.

**Table 2:** Behavioral intention to use according to the perceptions of the respondents.

Behavioral intention to use		Agree (n=256)			Disagree (n=16)			*P* value^a^
		Agree, n (%)	Disagree, n (%)	Agree, n (%)		Disagree, n (%)		
**Perceived usefulness**								
	Using SINADEF^b^ avoids falsifying a death certificate	220 (97.3)	36 (78.3)	6 (2.7)		10 (21.7)	<.001	
	Using SINADEF reduces the risk of errors in death certificates	221 (97.8)	35 (76.1)	5 (2.2)		11 (23.9)	<.001	
	Using SINADEF allows me to produce a death certificate in less time than doing it in paper format	207 (97.6)	49 (81.7)	5 (2.4)		11 (18.3)	<.001	
**Perceived ease of use**								
	I think SINADEF is easy to use.	236 (97.1)	20 (68.9)	7 (2.9)		9 (31)	<.001	
	It’s easy to get a password to use SINADEF.	148 (96.1)	108 (91.5)	6 (3.9)		10 (8.5)	.11	
	It’s easy to get technical support from SINADEF when you need it	93 (95.9)	163 (93.1)	4 (4.1)		12 (6.9)	.36	
	It’s easy to recover the SINADEF password when you forget it or it’s blocked	129 (95.6)	127 (92.7)	6 (4.4)		10 (7.3)	.25	
**Subjective norm**								
	I have to use SINADEF to certify deaths because it’s already established that way	208 (94.5)	48 (92.3)	12 (5.5)		4 (7.7)	.54	
	The authorities of my hospital and the Ministry of Health could sanction me if I do not use SINADEF to certify deaths	81 (93.1)	175 (94.6)	6 (6.9)		10 (5.4)	.63	
	I have to use SINADEF because everyone already uses it to certify deaths	195 (95.6)	61 (89.7)	9 (4.4)		7 (10.3)	.07	
**Image**								
	I feel comfortable with information and communication technologies	233 (95.5)	23 (82.1)	11 (4.5)		5 (17.9)	.004	
	I consider myself a person open to change	254 (94.8)	2 (50)	14 (5.2)		2 (50)	.02^c^	
	I have good adaptability	245 (94.6)	11 (84.6)	14 (5.4)		2 (15.4)	.14	
**Job relevance**								
	When SINADEF is implemented, the causes of death of the population will be known in a timelier manner	237 (95.9)	19 (76)	10 (4.1)		6 (24)	<.001	
	The use of SINADEF will help the directors or managers of health organizations make decisions	203 (96.7)	53 (85.5)	7 (3.3)		9 (14.5)	.001	
	If SINADEF is consolidated, it will be possible to quickly consult the data of the deceased	250 (96.2)	6 (50)	10 (3.8)		6 (50)	<.001	
**Output quality**								
	The SINADEF death certificate is of higher quality than the paper format	224 (87.5)	7 (43.8)	32 (12.5)		9 (56.3)	<.001	
**Results demonstrability**								
	I can easily communicate the results of my experience using SINADEF	224 (87.5)	7 (43.8)	32 (12.5)		9 (56.3)	<.001	
	For me, the results of using SINADEF will be visible	217 (84.8)	7 (43.8)	39 (15.2)		9 (56.3)	<.001	

^a^Chi-square test was performed.

^b^SINADEF: National Death Information System.

^c^Fisher exact test was performed.

### Multivariate Analysis

Three models were tested that had perceived usefulness as a dependent variable, corresponding to the 3 perceptions that evaluated perceived usefulness: (1) SINADEF avoids death certificate forgery, (2) SINADEF reduces the possibility of errors, and (3) SINADEF is faster than filling out paper forms. We use as perceived usefulness predictors the perceptions of the variables proposed in the TAM2 model: subjective norm, image, job relevance, output quality, and results demonstrability. The first model reached an overall percentage of correctly classified cases of 84.2% and found subjective norm, image, job relevance, and results demonstrability as predictors significantly associated with perceived usefulness but did not find perceived ease of use as a variable significantly related to perceived usefulness. The second model reached a global percentage of correctly classified cases of 88.2% and found perceived ease of use, subjective norm, image, and job relevance as predictors of perceived usefulness. The third model reached a global percentage of correctly classified cases of 82.7% and, in addition to perceived ease of use, only found job relevance and results demonstrability as perceived usefulness predictors. The second model was chosen because it showed the highest percentage of prediction and included 3 of the 5 variables proposed in the TAM2 model as significant predictors of perceived usefulness. The second model was based on the perception that SINADEF reduces the possibility of making errors when making a death certificate as an indicator of perceived usefulness. It was found that they were significantly associated with perceived usefulness: subjective norms such as the belief that SINADEF should be used because everyone already uses it to certify deaths (odds ratio [OR] 2.407, 95% CI 1.008-5.75; *P*=.048); image, the perception of being comfortable with ICT (OR 5.363, 95% CI 1.886-15.255; *P*=.002); job relevance such as the perception that the use of SINADEF will help health directors to make decisions (OR 4.49, 95% CI 1.978-10.2; *P*<.001); and perceived ease of use as the perception that SINADEF is easy to use (OR 18.95, 95% CI 6.634-54.156; *P*<.001). The model reached a predictive ability of 88.2% (see Table 3).

**Figure 2:**
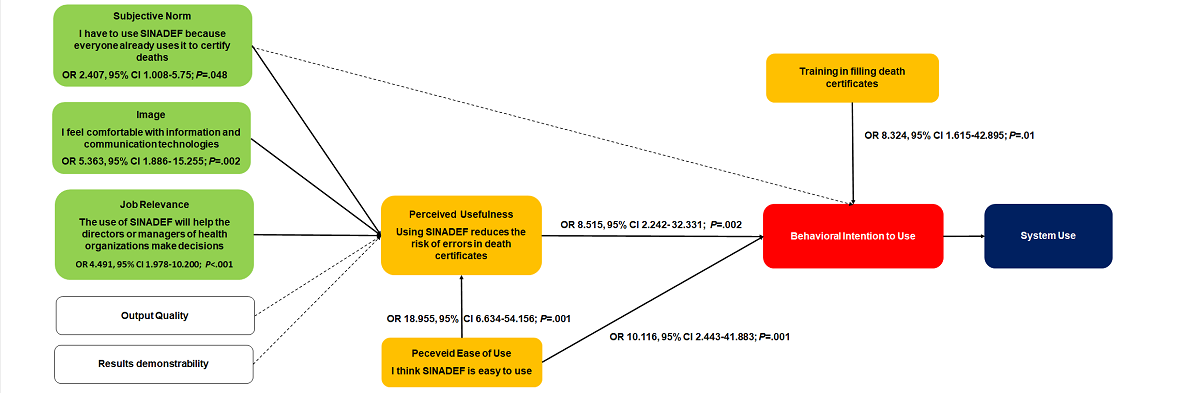
Summary of SINADEF behavioral intention to use predictors in the logistic regression models used. OR: odds ratio; SINADEF: National Death Information System.

The final model found the following statistically associated predictors with behavioral intention to use: perceived usefulness, measured by the belief that SINADEF reduces the risk of error in death certificate (OR 8.515, 95% CI 2.242-32.3; *P*=.002) and perceived ease of use, due to the belief that SINADEF is easy to use (OR 10.116, 95% CI 2.443-41.883; *P*=.001). Also included in this model was the variable training in the filling of death certificates, a variable external to the TAM2 model but which had a very important contribution (OR 8.324, 95% CI 1.615-42.895; *P*=.01). The final model reached a predictive ability of 95.6% (see Table 4 and Figure 2).

**Table 3:** Logistic regression of perceived usefulness predictors.

Predictor	*β*	Odds ratio (95% CI)	*P* value
Subjective norm: I have to use SINADEF^a^ because everyone already uses it to certify deaths	.879	2.407 (1.008-5.75)	.048
Image: I feel comfortable with information and communication technologies	1.502	5.363 (1.886-10.2)	.002
Job relevance: The use of SINADEF will help the directors or managers of health organizations make better decisions	1.045	4.491 (1.978- 5.931)	<.001
Perceived ease of use: I think SINADEF is easy to use	2.942	18.955 (6.634-54.156)	<.001
Constant	–10.153	0.000	<.001

^a^SINADEF: National Death Information System.

**Table 4:** Logistic regression of behavioral intention to use predictors.

Predictor	*β*	Odds ratio (95% CI)	*P* value
Perceived usefulness: Using SINADEF^a^ reduces the risk of errors in death certificates	2.142	8.515 (2.242-32.331)	.002
Perceived ease of use: I think SINADEF is easy to use	2.314	10.116 (2.443-41.883)	.001
External variable: Training in filling death certificate	2.119	8.324 (1.615-42.895)	.01
Constant	–10.313	0.000	<.001

^a^SINADEF: National Death Information System.

## Discussion

### Principal Results

Although it may seem that the implementation of an ICT project will be successful, this is not always the case. Projects can be successful, considering their management (scope, costs, and deadlines) from their gestation to their implementation [25]. However, in the case of ICT projects in health, their final adoption will also depend on the beliefs of health personnel, in which the real barriers and facilitators for their use can be identified [16]. SINADEF has contributed to improving the coverage [6] and the quality of information on causes of death [5]. However, until the end of 2017, its use rate was only 50%; therefore, it was pertinent to evaluate the intention of system use.

Most of the physicians interviewed considered themselves open to change. They believed that SINADEF would allow them to quickly consult the data of deceased people or that they could adopt innovative methods (95% or more). However, more than half of them did not agree with the idea that if they do not use the SINADEF, they should be sanctioned (68%) or that it is easy to obtain technical help when they have problems with the system (64%). Bivariate analysis revealed that perceived ease of use, measured by the belief that SINADEF is easy to use, was significantly associated with behavioral intention to use (*P*<.001). Perceived ease of use was measured on 4 propositions: the system is easy to use, it is easy to get a password, it is easy to get technical support, and it is easy to recover a password. Only the belief that the system is easy to use had a degree of agreement of 89.3%, while the other proposals had an agreement of less than 60%. Furthermore, in the bivariate analysis, only the perception that the system is easy to use was associated with behavioral intention to use; therefore, it was also part of the final logistic regression model to predict behavioral intention to use. In turn, perceived usefulness was measured with any of 3 ideas—SINADEF avoids falsifying a death certificate, fewer errors are made with SINADEF when creating a death certificate, or a death certificate is produced faster with SINADEF—and it was found to be associated with behavioral intention to use (*P*<.001). In addition, most or all the image, job relevance, output quality, and results demonstrability proposals were significantly associated with behavioral intention to use (*P*<.05).

In the multivariate analysis, among the 3 propositions that measured perceived usefulness, the proposition “using SINADEF reduces the risk of errors in death certificates” had the highest predictive capacity (88.2%). Subjective norm measured as “I have to use the SINADEF because everyone already uses it to certify deaths” (OR 2.4, 95% CI 1.0-5.8; *P*=.048), image measured as “I feel comfortable with technology” (OR 5.4, 95% CI 1.9-10.2; *P*=.002), job relevance measured as “the use of SINADEF will help directors or managers of health organizations to make decisions” (OR 4.5, 95% CI 2.0-5.9; *P*<.001), and perceived ease of use measured as “I believe that SINADEF is easy to use” (OR 19, 95% CI 6.6-54.2; *P*<.001) were significantly associated with perceived usefulness. In the final model, we found a significant association between perceived usefulness measured as “using SINADEF reduces the risk of errors in death certificates” (OR 8.5, 95% CI 2.2-32.3; *P*=.002), perceived ease of use measured as “I think that SINADEF is easy to use” (OR 10.1, 95% CI 2.4-41.9; *P*=.001), and training to fill out the death certificate (OR 8.3, 95% CI 1.9-42-9; *P*=.01), with behavioral intention to use. The correct classification of cases by the final model was 95.6%.

### Limitations of This Study

A limitation of this study is the selection of the participating physicians. The sample was made up of physicians who responded to an invitation sent to the email registered in SINADEF, and the response rate was less than 2%. This is because most physicians did not read the messages because they have an email account assigned by the institution where they work, which they usually do not use, or because the message went to the “spam” folder. Regarding the participants, there is a probability that they have incurred a social desirability bias. Despite this, we believe the sample to be representative of physicians in terms of graduation from universities in the country, specialties, sex, and age. In addition, the sample size was adequate for carrying out the proposed statistical models. Another aspect to consider is that a significant proportion of the surveys was completed in 2020, the initial year of the COVID-19 pandemic in Peru, in which SINADEF played an important role in monitoring mortality in Peru, and it is possible that this factor, not measured in this study, influenced the favorable opinion of physicians toward intention to use. It is also possible that the proposals to be accepted or rejected by the participants do not represent all the factors that may be linked to the process of the medical certification of deaths in Peru. It should also be considered that, as in all observational studies, it should not be inferred that there is a causal relationship between perceived usefulness and perceived ease of use and training with behavioral intention to use but only an association between them. This study also did not evaluate the performance or the real and objective effectiveness of the technology, but only the beliefs of the users about its usefulness that will motivate its future use and its final adoption.

### Comparison With Prior Work

There is a need to institutionalize SINADEF to intensify the process of improving the coverage and quality of information on causes of death. The use of paper forms, from the logistics of their supply to the procedures that ensure their entry into a database, can be a barrier to improving death certification [6]. This situation was verified during the COVID-19 pandemic by observing that the use of paper forms to certify a death decreased the probability that the data would enter the mortality database [9]. In addition, considering that there are 18 mortality information subsystems in Peru, most of which use paper forms and none of which have complete information [26], the consolidation of SINADEF as the main information system would reduce the fragmentation of currently existing data.

The usefulness that SINADEF has shown for mortality surveillance during the COVID-19 pandemic is another aspect that highlights the importance of contributing to its institutionalization. During the COVID-19 pandemic, SINADEF was used in several studies [10-12,27] as an important source of information to document excess mortality from all causes in Peru and was recognized as the most reliable way to measure the severity and the impact of COVID-19 on the population. With the support of SINADEF, an excess mortality of 371.9 per 100,000 inhabitants in 2020 was documented in Peru [28], and in general, all the researchers agreed in highlighting the importance of strengthening the mortality documentation system in Peru.

We have not identified studies of the application of TAM to evaluate acceptability, specifically in mortality information systems, but there are several publications that have studied the technological acceptance of health information systems that include electronic medical records or electronic health records. As in other studies, we found that perceived usefulness and perceived ease of use are powerful predictors of the intention to use SINADEF. These findings are consistent with most of the previous research synthesized in systematic reviews. Gagnon et al [29] carried out a systematic review of the factors that influence the adoption of ICT in health and found that the most common direct determinants of adoption were usefulness and ease of use. Later, Rahimi et al [21] reviewed 134 publications on the use of TAM to find out the perceptions of users of health information systems as predictors of the use of technology. Although the reference framework used or the methods for analyzing the results may differ, most of the studies reviewed agree in confirming that the perceived usefulness and the perceived ease of use are important predictors of the intention to use the technology.

In our study, the direct predictors of behavioral intention to use were perceived usefulness, perceived ease of use, and training in filling out death certificates, and the predictors of perceived usefulness were subjective norm, image, and job relevance. When reviewing the predictor variables of behavioral intention to use, in the cases under study, considerable heterogeneity was observed. Some studies found only 1 primary variable from TAM, such as perceived usefulness or perceived ease of use, associated with intention to use [30,31], and other studies reported subjective norm or job relevance directly related to behavioral intention to use, without being intermediated by perceived usefulness [30-32]. There are also studies that describe predictors of perceived ease of use, such as job relevance, management support, and training or computational self-efficacy [33,34], and some others found that age, sex, or clinical specialty were predictors of both perceived usefulness and perceived ease of use [31]. This variability supports the idea of contextualizing the results of each study not only referring to the type of technology used but also to the organizational culture in which the technology will work.

In our study, subjective norm (“I use SINADEF because everyone already uses it”), image (“I feel comfortable with technology”), and job relevance (“SINADEF helps managers to make decisions”) were predictors of perceived usefulness. This is partially consistent with studies reporting peer influence [35] or computational self-efficacy as perceived usefulness predictors [32,34]. Perceived usefulness measured by the idea that the system helps reduce errors is a performance indicator. In this regard, a study [36] found that improvements in performance are related to the intentions of health professionals to use electronic medical record systems.

In our perceived ease-of-use study, measured in its simplest and most direct way, “SINADEF is easy to use” was a strong and significant determinant of physicians’ intention to use SINADEF and influenced their perceived usefulness. However, the physicians did not agree with the perceived ease of use propositions that “it’s easy to obtain a username and password” or that “it’s easy to obtain technical support,” and indeed, these were not significantly related to the intention to use. In this regard, Boonstra and Broekhuis [37] reported that the lack of technical support to address problems that arise during system operation is a barrier to the adoption of the electronic medical record.

Training in filling out death certificates had a significant direct influence on behavioral intention to use. In this regard, the implementation of SINADEF was accompanied by a training process both in the competence to identify the cause of death and the technical management of the computer application. It is recognized that training to improve physicians’ knowledge regarding the proper filling of death certificates will improve the usability of mortality statistics. This study demonstrated that this activity is a direct predictor of the use of SINADEF in Peru [38]. This is consistent with the identification of the lack of technical knowledge and insufficient skills for the management of ICTs as a barrier to the adoption of technologies by becoming a source of resistance that hinders their adoption [37].

### Conclusions

According to our study, it seems that the intention to use SINADEF is related to the perception that it is an easy-to-use system, it is widely accepted, it improves the performance of the physicians who use it, and it helps to manage health services. Additionally, training in the filling of death certificates plays an important role in the intention to use the system. It also informs ICT decision-makers of, for example, opportunities for improvement to address possible barriers that may limit the sustainability of the system, such as deficiencies in technical support and in the timely resolution of emerging problems. This study provides important knowledge based on the opinion of physicians on the intention to use SINADEF, which should effectively contribute to its institutionalization in the country.

## Abbreviations

ICT: information and communication technology
OR: odds ratio
SINADEF: National Death Information System
TAM: Technology Acceptance Model
